# Abalone‐Inspired Adhesive and Photo‐Responsive Microparticle Delivery Systems for Periodontal Drug Therapy

**DOI:** 10.1002/advs.202202829

**Published:** 2022-08-30

**Authors:** Chuanhui Song, Danqing Huang, Cheng Zhao, Yuanjin Zhao

**Affiliations:** ^1^ Department of Rheumatology and Immunology Institute of Translational Medicine The Affiliated Drum Tower Hospital of Nanjing University Medical School Nanjing 210002 China; ^2^ Oujiang Laboratory (Zhejiang Lab for Regenerative Medicine, Vision and Brain Health) Wenzhou Institute University of Chinese Academy of Sciences Wenzhou Zhejiang 325001 China; ^3^ Chemistry and Biomedicine Innovation Center Nanjing University Nanjing 210023 China

**Keywords:** antibacterial microparticles, bioinspired materials, drug deliveries, microfluidics, particles, periodontitis

## Abstract

Antibiotics provide promising strategies for treating periodontitis, while their delivery and controllable release with desired oral retention remain challenging. Here, inspired by the unique suction‐cup structures of abalones, a novel adhesive and photo‐responsive microparticle (MP) delivery system is developed to treat periodontitis through microfluidic electrospray technology. Such MPs are generated by quickly ionic cross‐linking of sodium alginate together with photo‐curing of poly(ethylene glycol) diacrylate of the distorted microfluidic droplets during their high‐speed dropping into calcium chloride solution. Attributing to their unique concave structures, the abalone‐inspired MPs exhibit desired underwater adhesion ability and stability under running water. In addition, due to the loading of antibiotics minocycline hydrochloride and near‐infrared (NIR)‐responsive black phosphorus during their fabrication, the resultant MPs can not only eradicate bacteria directly, but also realize a controllable and effective drug release upon NIR irradiation. Based on these features, it is demonstrated from in vivo periodontitis that the abalone‐inspired MPs are firmly adhesive and can controlled‐release drugs on the tooth, and thus have outstanding antibacterial efficacy against *Porphyromonas gingivalis*. These results indicate the particular values of the abalone‐inspired MPs for oral‐related disease treatment.

## Graphical abstract

By encapsulating the black phosphorus and minocycline hydrochloride, the abalone‐inspired microparticles with the enhanced adhesive ability and controllable drug release capacity can realize antibacterial photothermal therapy on the tooth.

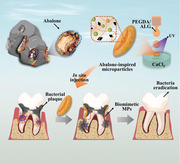

## 1 Introduction

As the primary reason for tooth loss in adults, periodontitis has now been considered the leading prevalent oral disease worldwide, causing humans unbearable suffering.^[^ ^1^ ^]^ Since periodontitis is initially caused by bacterial infection, the traditional surgical treatments, including scaling and root planning, cannot effectively eliminate the periodontal pathogens.^[^ ^2^ ^]^ Meanwhile, the surgical process would bring patients fear and discomfort.^[^ ^3^ ^]^ As an alternative, antibiotics, such as minocycline,^[^ ^4^ ^]^ metronidazole,^[^ ^5^ ^]^ and tinidazole,^[^ ^6^ ^]^ have shown promising potential in treating periodontitis.^[^ ^7^ ^]^ Especially by simply adding these antibiotics to ointments and applying the resultant ointments to the target area, the release of antibiotics would kill the pathogens effectively, bringing about complete inflammatory eradication.^[^ ^8^ ^]^ Despite with many successes of the antibiotics additive ointments in dental preparations, like the commercial production of minocycline ointments, which are applied in clinical practice worldwide, the continuous gingival crevicular fluid flow in the gingival sulcus would weaken the drug retention at the periodontitis site, leading to repeated administration of ointments and unavoidable drug waste.^[^ ^9^, ^10^ ^]^ Additionally, the passive release of actives from these ointments has also resulted in limited local antibiotics concentration and low therapeutic efficiency.^[^ ^11^, ^12^ ^]^ Therefore, a local drug delivery system with enhanced underwater adhesive property and controllable drug release capacity for treating periodontitis effectively is quite anticipated to develop.

**Figure 1:**
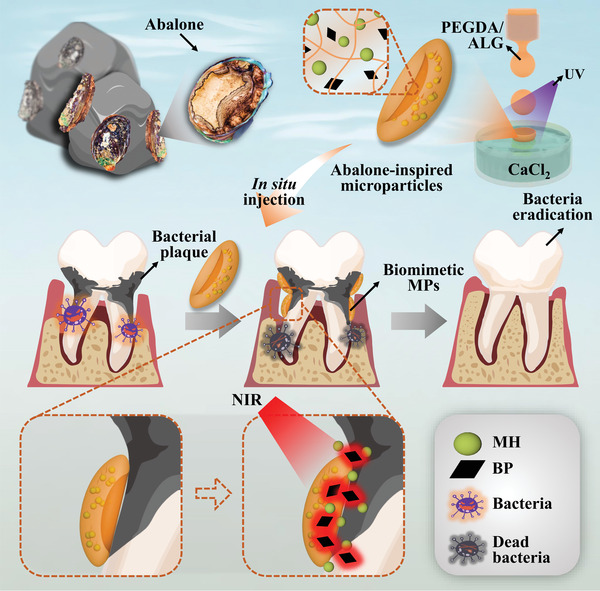
The graphical illustration of the abalone‐inspired microparticles' design, preparation process, and application. By encapsulating the BPs and MH, the microparticles with the enhanced adhesive ability and controllable drug release capacity could realize antibacterial photothermal therapy on the tooth.

In the paper, inspired by the unique proleg structures of abalones, we generate a novel adhesive and photo‐responsive microparticle (MP) delivery system to treat periodontitis through microfluidic electrospray technology (**Figure** **1**). Attributing its splendid micron‐scale maneuverability, microfluidic technology has drawn increasing attention to the fabrication of MPs for drug delivery systems.^[^ ^13^, ^14^, ^15^, ^16^ ^]^ With the addition of energy‐converting responsive materials during the fabrication processes, such as graphene oxide,^[^ ^17^, ^18^ ^]^ polydopamine,^[^ ^19^ ^]^ and black phosphorus (BP),^[^ ^20^, ^21^, ^22^ ^]^ these MPs can also be endowed with controllable drug release properties upon external stimuli like near‐infrared ray (NIR) irradiation.^[^ ^23^, ^24^, ^25^, ^26^, ^27^ ^]^ In contrast, various adhesive stratagems have been employed by natural creatures to guarantee their survival.^[^ ^28^, ^29^ ^]^ For instance, the suction‐cup‐structured prolegs of abalones feature with extraordinary adhesion capacity underwater, making them able to stick to rocks in the sea stably.^[^ ^30^ ^]^ These features provide many bio‐inspirations for creating adhesive matters. Thus, it is conceivable to develop a new functional MP delivery system by integrating the advantages of microfluidics, stimuli‐sensitive materials, and abalone‐mimic structures for periodontal drug therapy.

Herein, we fabricated the desired BP MPs with the abalone‐like adhesion and photo‐responsive release of minocycline hydrochloride (MH) via electrostatic spraying microfluidic strategy to treat periodontitis. Through the fast ionic cross‐linking of sodium alginate (ALG) and the sudden photo‐curing of poly(ethylene glycol) diacrylate (PEGDA), the high‐speed dropped microfluidic droplets first form the back of the disc in calcium chloride (CaCl_2_) solution and simultaneously solidified their concave under ultraviolet (UV) irradiation. It was found that compared with the medical ointment and spherical MPs, the disc‐like MPs displayed enhanced adhesive ability underwater. In addition, ascribing to the photothermal converting property of the additive BP, the resultant MPs can eradicate bacteria directly and realize a controllable and effective drug release upon exposure to NIR irradiation. This synergistic antibacterial function of our abalone‐inspired MPs makes them valuable effect in treating in vivo periodontitis. Thus, this research offers a distinctive candidate for designing a bio‐inspired MP‐based delivery system to treat oral‐related diseases.

## 2 Results and Discussion

**Figure 2:**
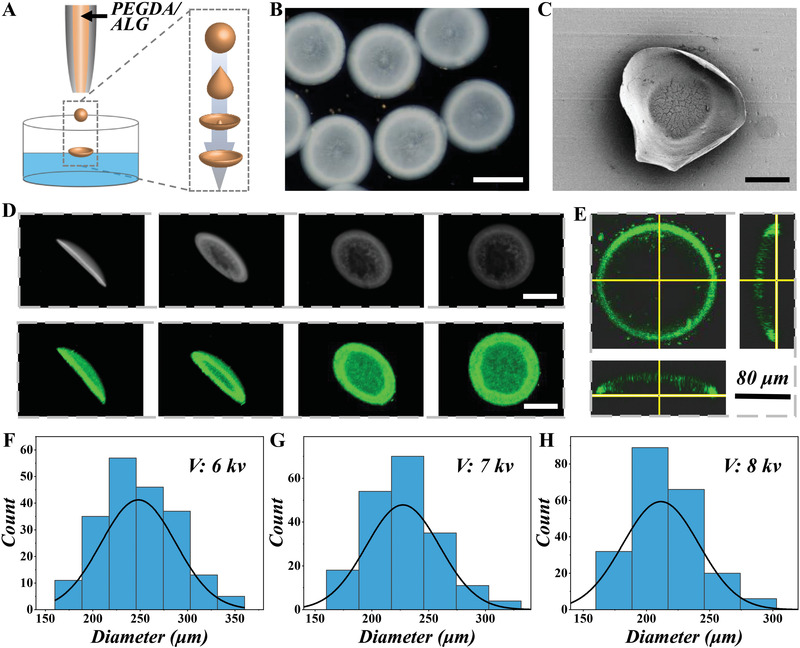
The preparation and characterization of the bio‐inspired MPs via microfluidic electrospray. A) The schematic diagram shows microparticles generation. B) The bright‐field microscopic pictures show the morphology of MPs. Scale bars = 200 µm. C) The scanning electron microscope images of the MP. Scale bars = 100 µm. D) The digital pictures and fluorescent images of an MP with different angles. Scale bars = 100 µm. E) The 3D confocal image and the cross‐sectional images of the FITC‐loaded MPs outline MPs' suction‐cup structure. Scale bars = 80 µm. F–H) The diameter distribution of microparticles under different voltages. F) 6 kv, G) 7 kv, and H) 8 kv.

In the typical experiment, ALG solution was mixed with PEGDA homogeneously as the precursor solution to generate abalone‐like MPs. The solution was controllably injected through a microfluidic device during the electrospray process. Under the high voltage electric field created through a direct current instrument, the mixed pregel solution overcame the surface tension and then turned into micro‐droplets while falling into the CaCl_2_ solution (**Figure** **2**A). The droplets then polymerized after the calcium cross‐linking of ALG followed by UV curing of PEGDA.^[^ ^31^ ^]^ The quick ionic cross‐linking of alginate and the photocuring of PEGDA without using organic solvents could retain the bioactivity of antibiotics. Also, compared with GelMA, the PEGDA can protect the MH from the enzyme in saliva. These two different curing methods imparted the MPs with two different compositions on two sides. When the droplet hits the solution under the electric field force, it can deform owing to the liquid surface tension, during which the solid calcium ALG forms the outer surface of the MPs. Subsequently, the UV‐polymerized PEGDA compartment formed the inner surface of the MP. The abalone‐like MPs can be obtained from the liquid surface tension and diverse curing methods (Figure 2B). The condition in the gingival sulcus is like the abalone's living environment. So our abalone‐inspired MPs focus on the shape‐mimicking of abalone and the situation where abalone stays. Also, it should be mentioned that the deformation process can emerge as the jellyfish‐shaped MPs also obtained in our experiment (Figure S1A, Supporting Information), which corresponds with previous research.^[^ ^32^ ^]^

Also, to confirm the inner and outer surface of the MPs, a scanning electron microscope was taken to observe their microsurface (Figure 2C), where the outer surface showed a morphology more ALG‐like solid formation, which conforms to the design feature (Figure S1B–D, Supporting Information). Next, to observe the size and shape of the MPs more intuitively, we used FITC‐IgG to characterize the MPs, which can emit green fluorescence and be detected under a fluorescence microscope (Figure 2D). As shown in Figure 2E, the disc and arch features of the MPs can be observed in the 3D confocal images and the cross‐sectional images, corresponding to our design. The unique shape of the MPs can impart them with adhesive features, making them conceivable to be applied in wetter environments like the oral cavity. To test the stability of the synthesis method, we measured the diameters of different MPs under different conditions. Results showed that the diameter of the MPs can be regulated from 380 to 200 µm by tuning the electric field between the microfluidic outer orifice and the surface of the collecting buffer (Figure 2F–H and Figure S2, Supporting Information). Meanwhile, the distance between the microfluidic outer orifice and the collecting buffer's surface can also affect the MPs' size. When the distance was too short, the pregel solution directly dropped into the CaCl_2_ solution and formed spherical particles (Figure S3A, Supporting Information). And with suitable distance and voltage, the designed bio‐inspired MPs can be fabricated (Figure S3B, Supporting Information). These results showed that we synthesized the abalone‐like MPs successfully, and our microfluidic electrospray fabricating method can achieve mass, stable, and uniform production.

**Figure 3:**
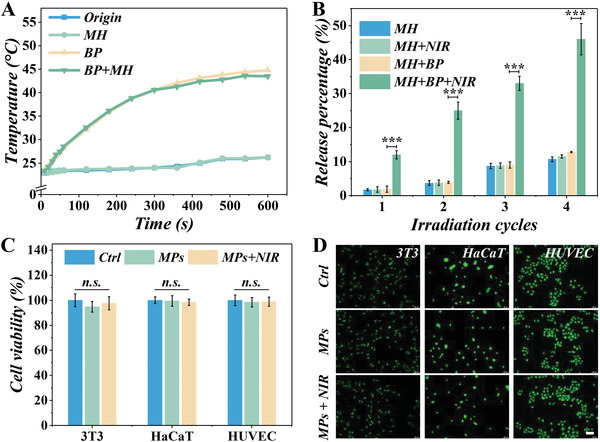
The in vitro drug release profile and biocompatible experiment. A) The temperature change profiles of the solution containing particles. B) The MH concentration after laser irradiation. C) The CCK‐8 results of the HaCaTs, 3T3 cells, and HUVECs. D) The Calcein‐AM staining of corresponding cells after 24 h incubation. Scale bar = 100 µm. (*n* = 3. ***p < 0.001).

As BP own the photothermal effect, we take BP as the energy‐converting responsive materials to endow MPs with photothermal effect via doping BP in the pregel to fabricate MPs. To further investigate the photothermal effects of the MPs, the temperature variations were obtained by a thermometer during laser irradiation. After 5 min of irradiation, the temperature can reach 42 °C (**Figure** **3**A), and the temperature change was proportional to the laser power (Figure S4A, Supporting Information). The photothermal conversion efficiency of MPs was about 35.2% after calculation, which is consistent with the previous BP report.^[^ ^33^ ^]^ The results indicated that the MPs had good photothermal conversion efficiency and could realize antibacterial photothermal therapy and promote drug release, as the high temperature can enhance the molecular movement of the drug.^[^ ^20^ ^]^ To analyze the release kinetics of MH, we detected the concentration of MH in solutions containing MPs. As shown in Figure 3B, the MH concentration increased tremendously with prolonged laser irradiation; the MH was released about 45% from the MPs after four irradiation cycles, indicating that the NIR laser‐induced hyperthermia can trigger drug release effectively. To test the biosafety of the drug‐loaded MPs (load with BP and MH), several cell lines were employed, including 3T3 cells (the murine fibroblast cells), HaCaTs cells (the human normal epithelial cells), and HUVECs cells. After the co‐incubation with the drug‐loaded MPs for 24 h, respectively, the cell counting kit‐8 (CCK‐8) experimental results illustrated good biological cell activity of the HaCaTs, 3T3 cells, and HUVECs cells (Figure 3C), indicating the outstanding biocompatibility of the MPs. Furthermore, to observe the cell condition directly, the Calcein‐AM was used to stain the living cells after the 24 h incubation. The green fluorescence signals in the MPs and MPs + NIR groups were nearly equal to the control group (Figure 3D and Figure S4B, Supporting Information), demonstrating that the cells were sound with MPs and NIR. All those data indicated that the drug‐loaded MPs could achieve the NIR‐response drug release and own good biocompatibility, showing good bio‐application potential.

**Figure 4:**
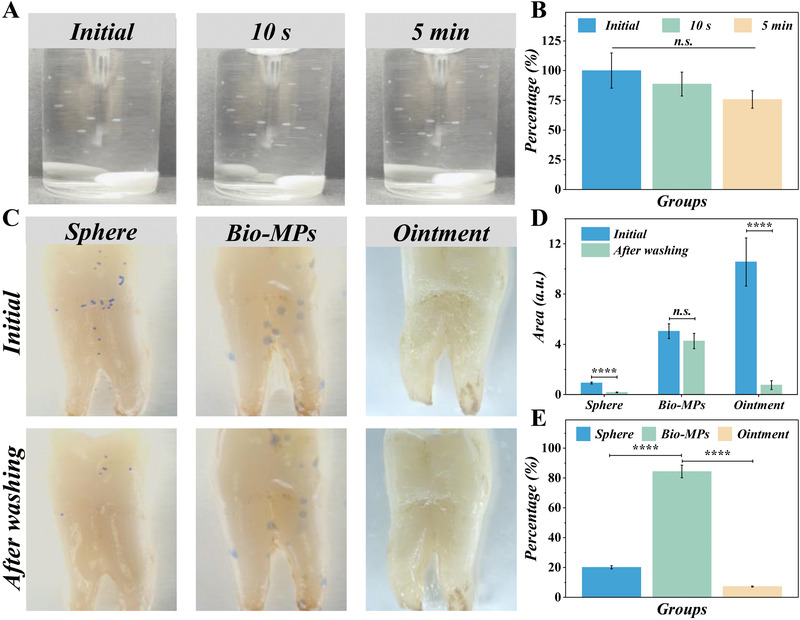
Analysis of the adhesive ability of the abalone‐inspired MPs. A) The bio‐inspired MPs in a vial after stirring for different times. B) The quantification of numbers of MPs during stirring. C) The pictures of sphere MPs, bio‐inspired MPs, and ointment attach to the teeth before (up) and after (down) water swash. D) The statistical analysis of the area of spherical MPs, ointment, and abalone‐shaped MPs. E) The percentage of the cover region after washing, the percentage was calculated from the formula (percentage = area_after washing_/area_initial_ × 100%). (*n* = 3. ****p < 0.0001).

Before the antibacterial application of the MPs on teeth, the adhesion and retention capacity of the MPs in the gingival sulcus are first investigated. The diameter may influence the adherence ability of MPs, so we tested the remaining rate of MPs after washing. The results showed that the diameter had no significant impact on adhering ability above or below 300 µm (Figure S5A–C, Supporting Information). The prepared MPs were dispersed for 5 days to test the agglomeration. The results showed the MPs had no apparent aggregation after 5 days of storage (Figure S5C, Supporting Information). To further test the adhesive ability under underwater conditions, we applied the abalone‐shaped MPs onto the vial wall to simulate the natural environment underwater. After stirring for 5 min, about 80% of the bio‐MPs were still attached to the vial (**Figure** **4**A,B). To further mimic the natural oral cavity, the isolated tooth was taken, and a continuous water current was added after different particles were attached to the tooth's surface. The normal spherical MPs and clinically employed ointment were set to compare with the abalone‐shaped MPs. The different MPs and ointment were applied to the tooth surface, after which the pictures were taken before and after a continuous water current as “initial” and “after washing.” The area of MPs or ointment cover was analyzed to compare the difference between each other. Compared with the sphere and ointment, the bio‐inspired MPs (Figure 4C) showed more retention proportion after water irrigation. The spherical MPs could hardly attach to the teeth' surface, and a water current could wash them away easily. The ointment was also washed out, though their origin area was more than the others. The bio‐inspired MPs characterized enhanced adhesive ability due to the unique structure, showing more cover region after water washing (Figure 4D,E). All those results showed that the bio‐inspired MPs had a great potential in attaching to the tooth in the oral cavity and treating tooth plaque.

**Figure 5:**
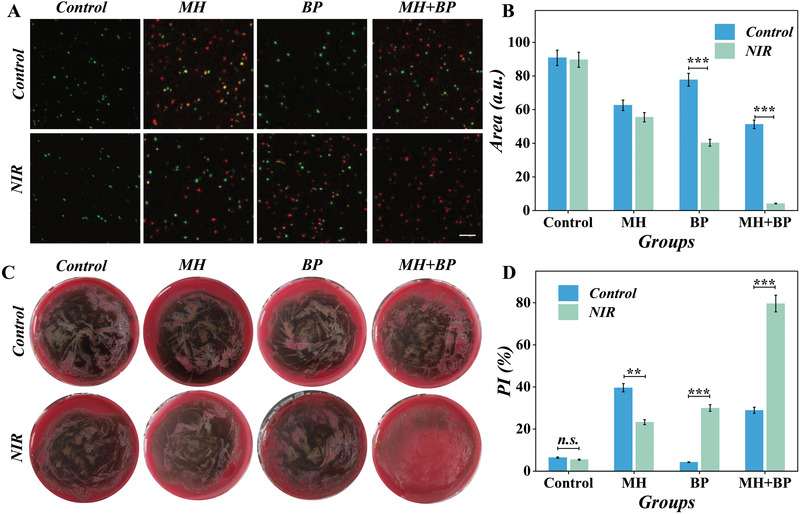
The antibacterial of microparticles in vitro. A) The fluorescent images of bacteria after Syto/PI staining. B) The quantitative analysis of fluorescent images in (A) through Fiji. C) The images of bacteria colony formation after different treatments. D) The quantitative analysis of colony formation. (*n* = 3. ***p < 0.001).

With the MH and BPs addition, the bio‐inspired MPs drug delivery system accompanied with laser irradiation demonstrated excellent antibacterial properties toward *Porphyromonas gingivalis* (*P. gingivalis*). To directly observe the state of bacterial, live/dead staining was conducted after various treatments. The fluorescent images illustrated that the proportion of deadly bacteria improved dramatically after synergistic treatment of MH and NIR‐excited BPs; the red fluorescence signal significantly increased, and the green fluorescence became weak (**Figure** **5**A). The quantitate data also proved the results (Figure 5B). Also, the BP group with NIR irradiation showed antibacterial ability. Meanwhile, to observe the growth of *P. gingivalis*, a colony formation test was conducted in the solid culture medium. We checked the clone formation every other day and after 10 days of culture,^[^ ^34^ ^]^ the MN and BP‐encapsulated bio‐inspired MPs accompanied with NIR irradiation showed minimal clone formation, implying inhibited bacterial growth (Figure 5C,D). The NIR with tissue penetration ability can reach deep tissue through soft and hard tissue for bone repair and tumor therapy with minimal side effect to healthy tissue.^[^ ^35^ ^]^ So, it is believed that the NIR can make sense in periodontal for therapeutic demand. Collectively, these data suggested that the MN and BP co‐loaded MPs with a NIR laser excitation could significantly inhibit bacterial growth, indicating their application potential in periodontal drug therapy.

**Figure 6:**
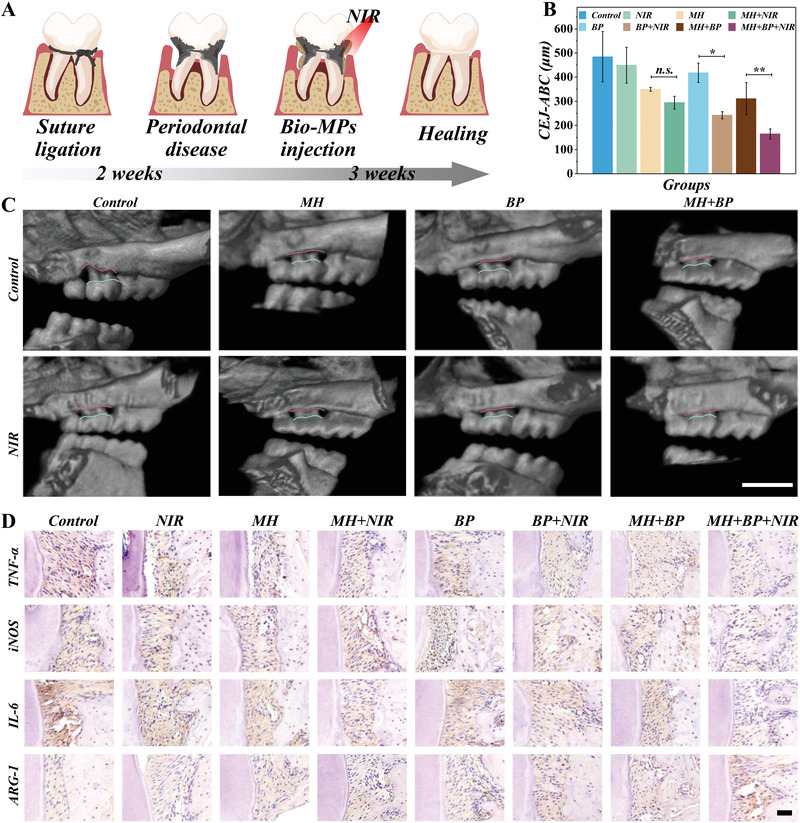
The in vivo therapeutic effect of the MPs against periodontitis. A) Scheme of the periodontitis model establishment and the treatment process. C) The 3D reconstructions of the maxillary molar area and B) the quantitative analysis of CEJ‐ABC. Scale bar = 1 mm. D) The IHC staining of the maxillary molar area. Scale bar = 50 µm. (*n* = 3. **p < 0.01, *p < 0.05).

To test the in vivo therapeutic effects, we established periodontitis animal models through the silk suture ligation method for 2 weeks, during which the cariogenic bacterial suspension was coated twice a week. After 2 weeks, the periodontitis model was confirmed by micro‐CT, where the local bone loss could be detected (**Figure** **6**A). Clinical symptoms like tooth loose and periodontal inflammation also can be detected in the mouse oral cavity. To evaluate the therapeutic effect, the MPs were gently placed into the gingival sulcus, followed by the NIR irradiation. The results were evaluated by clinical checks, such as the color and shape of the gingival and the micro‐CT manifestations after the therapeutic period. Through 3D reconstructions, the vertical spacing between the alveolar bone crest (ABC) of the alveolar and the tooth's cementoenamel junction (CEJ) was taken as a therapeutic index. Compared with other groups, bio‐inspired MPs with NIR irradiation showed a minimized distance and almost recovered to a healthy level (Figure 6B,C). The BP own abundant phosphorus, which may contribute to the bone repair during the healing period.^[^ ^36^ ^]^ Also, the antibacterial effect based on the bio‐inspired MPs with NIR irradiation eradiated the local pathogenic, reducing the inflammation and promoting bone repair. Immunohistochemical (IHC) staining was carried out to evaluate the angiogenesis and inflammation markers and explore the underlying tissue repair mechanisms. The tumor necrosis factor‐alpha (TNF‐*α*), together with inducible nitric oxide synthase (iNOS), interleukin‐6 (IL‐6), and arginase‐1 (ARG‐1), were selected to evaluate the inflammation states. As shown in Figure 6D, the treated group offered a reduced inflammatory environment, in which the TNF‐*α*, iNOS, and IL‐6 were dramatically diminished. Inversely, the ARG‐1, a marker of M2‐type macrophage, increased in the treated group, indicating the promotion of osteogenesis and inhibition of osteoclastogenesis. These results demonstrated that our NIR‐excited abalone‐shaped MPs could relieve the local inflammatory environment, thus accelerating the recovery of periodontitis (Figure 6D and Figure S7, Supporting Information).

**Figure 7:**
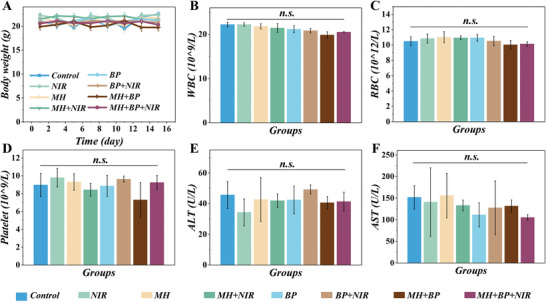
The biosafety analysis of the NIR‐exited MPs therapy in vivo. A) The body weight change profiles during the therapeutic period. B–F) The hematology results of mice after treatments. (n.s. = not significant).

To test the treatment biosafety, the body weight was measured during the period to reflect the usual status of mice. There was no apparent impact on the body weight (**Figure** **7**A). Also, the hematoxylin and eosin (H&E) staining of the maxillary molar area was demonstrated in Figure S6, Supporting Information. The hematology test also showed the biocompatibility of the treatments since the complete blood count was normal, and the renal and liver function exams were also in normal scope (Figure 7B–F and Figure S8, Supporting Information). Meanwhile, after treatments, the H&E check of the collected murine main organs showed no significant pathological changes in the bio‐inspired group (Figure S9, Supporting Information), indicating excellent biosafety. Therefore, these multifunctional MPs preliminarily exhibited particular potential in clinical practice.

## 3 Conclusion

In summary, inspired by the abalone's adhesive ability underwater, we proposed the abalone‐like MPs with strong adherence property toward the teeth for local drug delivery. Electrostatically driven microfluidic technology and the dual polymerization of ALG and PEGDA enabled bio‐inspired MPs' mass and uniform fabrication. Attributing to the unique structure, the MPs had a better adhesive ability underwater than traditional spherical MPs and clinically employed ointment. With the innate BP's photothermal ability, the controlled drug release can be achieved by NIR irradiation, and the BP and MH showed outstanding antibacterial performance in vitro. In animal models of periodontitis, the bio‐inspired MPs demonstrated an excellent antibacterial property, and the periodontal tissue showed relieving inflammation. Moreover, the clinical symptoms and manifestations of periodontitis were significantly reduced according to micro‐CT images. Therefore, the abalone‐like MPs exhibited outstanding adhesion capacity underwater and are expected to pave a new way in local drug delivery and periodontal therapy.

## 4 Experimental Section

### Materials

PEGDA (average Mn = 700), ALG (very low viscosity), and CaCl_2_ were purchased from Aladdin. Calcein‐AM/PI was from Beyotime. 2‐hydroxy‐2‐methyl‐1‐phenyl‐1‐propanone (HMPP) and 4% paraformaldehyde were obtained from Adamas‐Beta. CoraLite 488 conjugated IgG (H + L) (SA00013‐2) were purchased from Proteintech. The *P. gingivalis* was from the beijingbio.tech, and cultured with Columbia blood agar plates or modified brain heart infusion (BHI) broth under an anaerobic environment at 37 °C. The female C57BL/6 mice were purchased from the Qinglong mountain animal breeding field. The Animal Investigation Ethics Committee of The Affiliated Drum Tower Hospital of Nanjing University Medical School reviewed all animals' experimental protocols and care (No. 2019AE01012).

### Microfluidic Electrospray Device

The microfluidic device consisted of a syringe and a tapered glass capillary connected with a microtube. The capillary was tapered with an alcohol burner, followed by sand to reach an expected diameter.

### Microparticles Development

ALG (2%), PEGDA (25%), and HMPP (1%) were mixed to form the transparent and homogeneous origin liquid. Under the force of the high voltage‐induced electric field, the microdroplets hit the 2% CaCl_2_ forming the surface immediately, and UV light could solidify the PEGDA in the MPs to form a disc‐like shape. The size of MPs was analyzed under different voltages (U). To load the BPs and/or MH, the BP and MH (0.9%) (2%) were added to origin liquid to form a pregel under ultrasonic treatment, the final concentration were 0.9% m/v and 2% m/v, separately. The drug loaded MPs were fabricated followed the above method.

### Characterization

To gain a 3D reconstruction of MPs, the fluorescent IgG was added to the premixed liquid. To detect the fluorescence image of MPs directly, the confocal laser scanning microscope (Nikon A1) was employed. After being wholly solidified, a gradient dehydration method was used to obtain dried microspheres for the scanning electron microscope test.

### Photothermal Effect Test

After MPs solidified, they were added to a 96‐well plate. The different power NIR irradiated for other times, during which a thermal recorder recorded the temperature of the solution.

### Drug Release Test

A spectrometer detected the supernatant at 375 nm to measure the concentration of MH, with or without the laser irradiation, at different time points.

### Antibacterial Ability In Vitro

The antibacterial treatments were performed according to previous literature. Briefly, the bacterial suspensions were mixed with bio‐inspired MPs. Then, NIR (660 nm, 0.3 W cm^−2^) was utilized as a light source to illuminate the treated bacterial suspensions. After incubation for 4 h at 37 °C, the Syto/PI assay kit was used to detect the live/dead of *P. gingivalis*. The antibacterial experiments were quantitatively measured by the spread plate method. After the different treatments, the appropriate diluted bacterial suspensions were uniformly plated on standard TSB agar plates for *P. gingivalis*. The plates were incubated for 5 days to count the corresponding bacterial colonies.

### In Vivo Study of the Treatment of Periodontitis

For modeling of periodontitis, after mice were anesthetized, the maxillary second molar was tied by a 5‐0 silk ligature, and the fixed ligature was kept for 2 weeks. Meanwhile, the bacterial suspensions were instilled around the fixed ligature. After 2 weeks, the mice were tested with micro‐CT to conform to periodontitis. For treatment of periodontitis, the bio‐inspired MPs were placed into the gingival sulcus, followed by irradiation of laser for 5 min. After completing the corresponding experiments, all mice were euthanized at a specific time point.

### Histology Analysis

The mice tissues, including teeth, gingival, and jawbone, were collected to stain H&E. iNOS, TNF‐*α*, IL‐6, and ARG‐1 were selected to do IHC to evaluate angiogenesis and local inflammation. Also, the major organs were gathered to test system toxicity compared with healthy mice.

### Statistical Analysis

In this paper, all data were analyzed with GraphPad Prism. The data shown in the graph were expressed as mean ± SD (standard deviation). The sample size (*n*) for each statistical analysis is provided in the figure legends. The student's *t*‐test was used to calculate statistical differences between two groups, and a one‐way analysis of variance was used to compare differences between more than two groups. The value of *p* <0.05 was considered statistically significant.

## Conflict of Interest

The authors declare no conflict of interest.

## Author Contributions

Y.J.Z. conceived the idea and designed the experiment. C.H.S and D.Q.H wrote the paper. C.H.S. and C.Z. conducted the experiments and analyzed the data.

## Supporting information

Supporting Information

## Data Availability

The data that support the findings of this study are available from the corresponding author upon reasonable request.
