# Supplementary material for: Abalone‐Inspired Adhesive and Photo‐Responsive Microparticle Delivery Systems for Periodontal Drug Therapy
Source: Adv Sci (Weinh). 2022 Aug 30;9(30):2202829. doi: 10.1002/advs.202202829 (PMC9596845; doi:10.1002/advs.202202829)
Supplement: Supplementary file 1 — Supporting Information [file ADVS-9-2202829-s001.pdf]

ADVANCED SCIENCE
Open Access

Supporting Information

for *Adv. Sci.*, DOI 10.1002/advs.202202829

Abalone-Inspired Adhesive and Photo-Responsive Microparticle Delivery Systems for Periodontal Drug Therapy

*Chuanhui Song, Danqing Huang, Cheng Zhao and Yuanjin Zhao**

Supplementary Information

Abalone-inspired adhesive and photo-responsive microparticles delivery systems from microfluidic electrospray for periodontal drug therapy

*Chuanhui Song [a], Danqing Huang [a], Cheng Zhao [a], Yuanjin Zhao[a,b,c,]**

a. Department of Rheumatology and Immunology, Institute of Translational Medicine, The Affiliated Drum Tower Hospital of Nanjing University Medical School, Nanjing, 210002, China.

b. Oujiang Laboratory (Zhejiang Lab for Regenerative Medicine, Vision and Brain Health), Wenzhou Institute, University of Chinese Academy of Sciences, Wenzhou, Zhejiang 325001, China

c. Chemistry and Biomedicine Innovation Center, Nanjing University, Nanjing, 210023, China.

Email: yjzhao@seu.edu.cn

Supporting Figures:

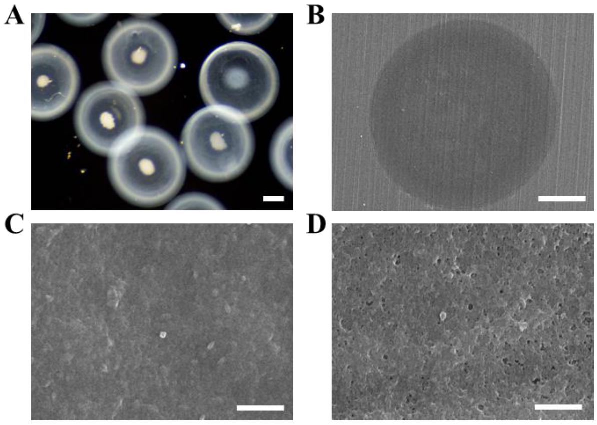

Figure S1. (A) Bright-field microscopic images of the jellyfish-shape MPs. (B-D) The SEM images of the MPs (B), the outer (C) and the inner surface (D) of the dual-composition MPs. Scale bars are 50 μm in (A) and (B), 100 nm in (C) and (D).

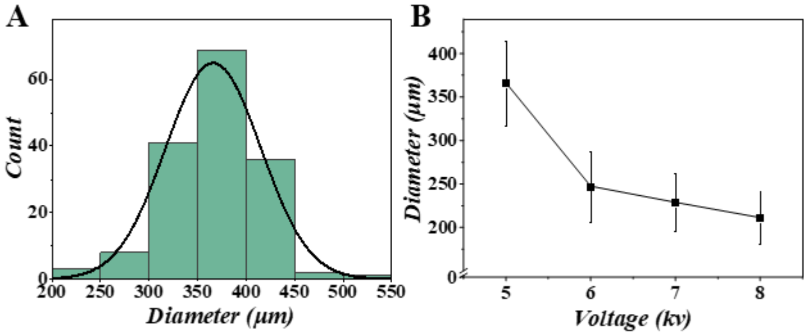

Figure S2. The analysis of microparticles' shape and size under different conditions. (A) The diameter of microparticles under 5 kv. (B) The statistical analysis of microparticles' size.

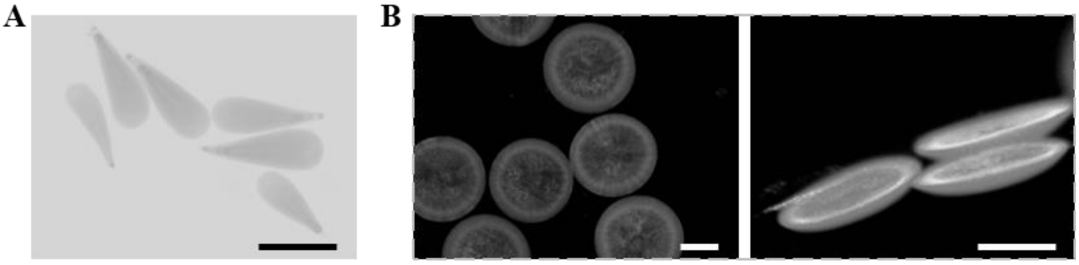

Figure S3. (A) The image of microparticles under short distance. Photographs of the particles in the tiled (B) and upright state (C). Scale bar is 100 μm.

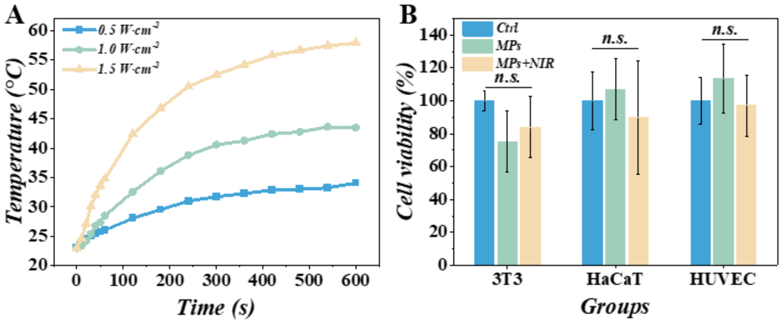

Figure S4. (A) The temperature change profile of bio-MPs under different power of NIR. (B) The quantitively analysis of Figure 3D.

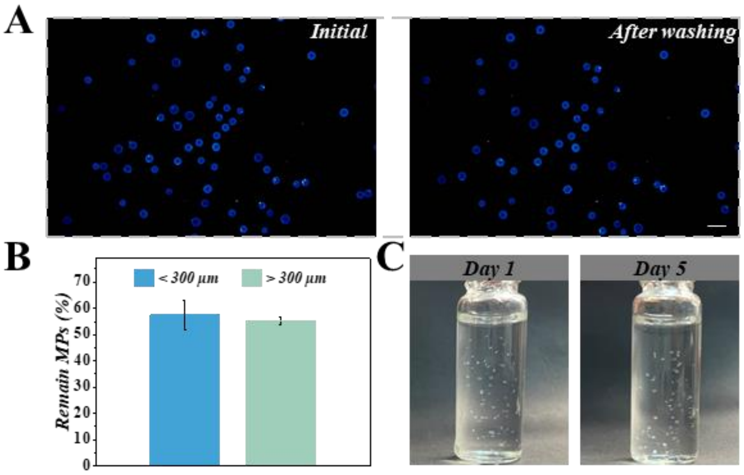

Figure S5. (A) The bio-inspired MPs before and after the water washing. (B) The quantification of MPs in (A), the remain percent was calculated through Number $_{(after\ washing)}$/Number $_{(initial)}$×100%. (C) The photo of MPs storage for 5 days. The photos were taken after resuspend.

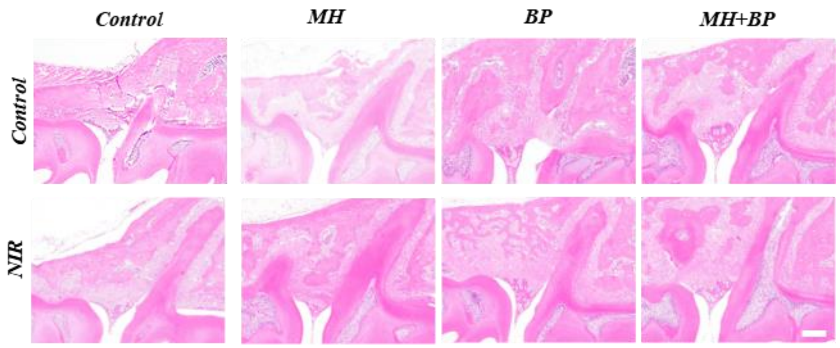

Figure S6. The H&E staining of the maxillary molar area after treatment. Scale bar = 200 μm.

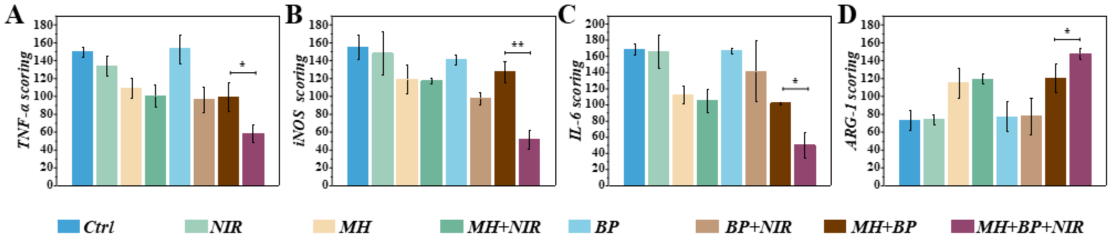

Figure S7. The quantitative analysis of IHC.

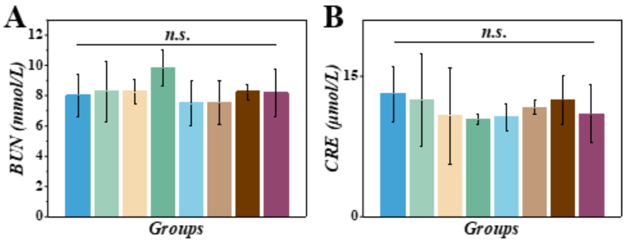

Figure S8. The renal function analysis of mice after treatments.

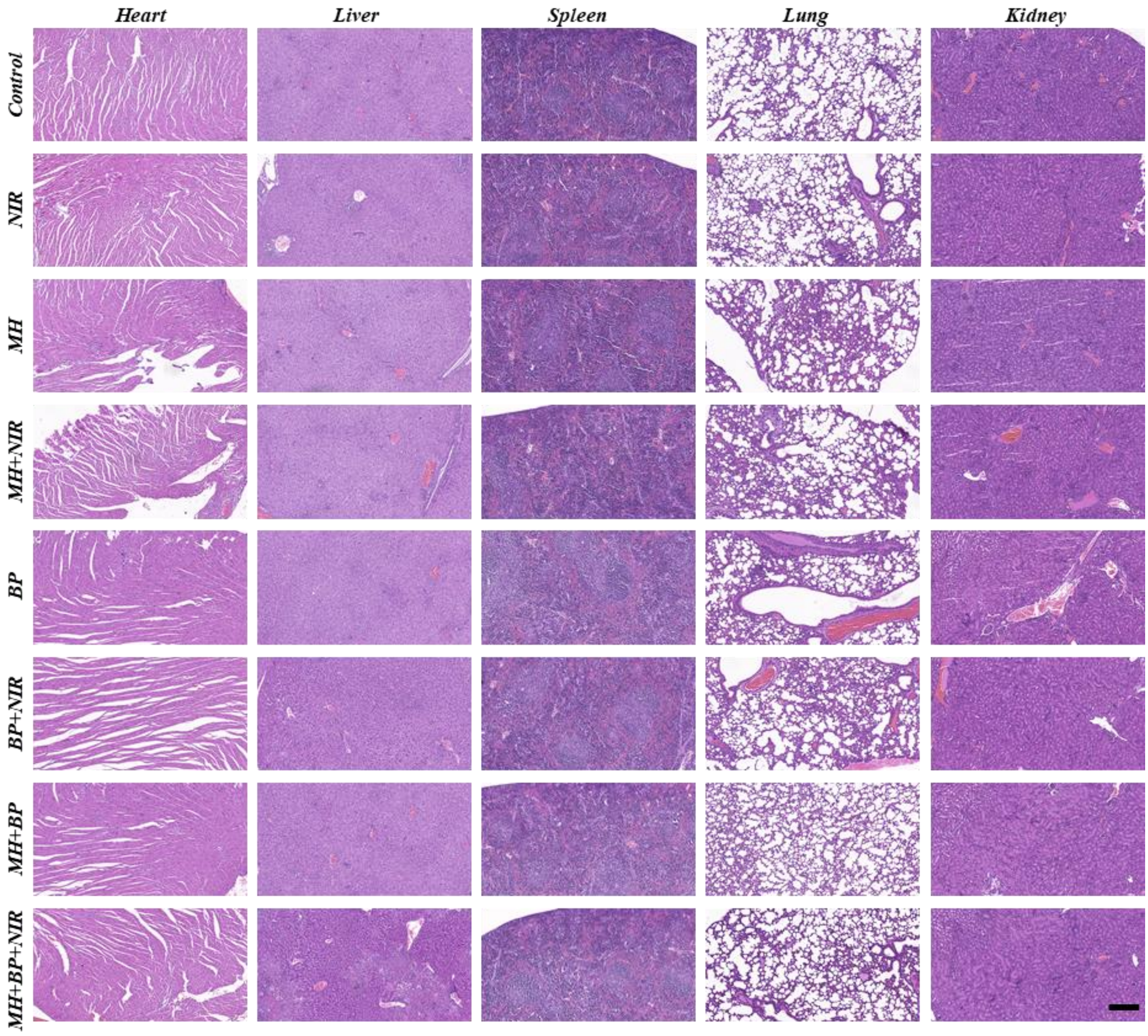

Figure S9. The hematoxylin & eosin (H&E) staining of collected organs embedded in paraffin. Scale bar:100 μm.
